# A stable lithium-rich surface structure for lithium-rich layered cathode materials

**DOI:** 10.1038/ncomms13598

**Published:** 2016-11-25

**Authors:** Sangryun Kim, Woosuk Cho, Xiaobin Zhang, Yoshifumi Oshima, Jang Wook Choi

**Affiliations:** 1 Graduate School of Energy, Environment, Water, and Sustainability (EEWS), Korea Advanced Institute of Science and Technology (KAIST), 291 Daehak-ro, Yuseong-gu, Daejeon 305-701, Republic of Korea; 2 KAIST Institute NanoCentury, Korea Advanced Institute of Science and Technology (KAIST), 291 Daehak-ro, Yuseong-gu, Daejeon 305-701, Republic of Korea; 3 Department of Chemical Science and Engineering, School of Materials and Chemical Technology, Tokyo Institute of Technology, 4259 Nagatsuta, Midori-ku, Yokohama 226-8502, Japan; 4 Advanced Batteries Research Center, Korea Electronics Technology Institute (KETI), 25 Saenari-ro, Bundang-gu, Seongnam 463-816, Republic of Korea; 5 School of Materials Science I, Japan Advanced Institute of Science and Technology (JAIST), 1-1 M1-61 Asahidai, Nomi 923-1292, Japan

## Abstract

Lithium ion batteries are encountering ever-growing demand for further increases in energy density. Li-rich layered oxides are considered a feasible solution to meet this demand because their specific capacities often surpass 200 mAh g^−1^ due to the additional lithium occupation in the transition metal layers. However, this lithium arrangement, in turn, triggers cation mixing with the transition metals, causing phase transitions during cycling and loss of reversible capacity. Here we report a Li-rich layered surface bearing a consistent framework with the host, in which nickel is regularly arranged between the transition metal layers. This surface structure mitigates unwanted phase transitions, improving the cycling stability. This surface modification enables a reversible capacity of 218.3 mAh g^−1^ at 1*C* (250 mA g^−1^) with improved cycle retention (94.1% after 100 cycles). The present surface design can be applied to various battery electrodes that suffer from structural degradations propagating from the surface.

## Abstract (web summary)

Surface modification of high-capacity lithium-rich layered oxides for improved capacity retention is an active area of battery materials research. Here authors demonstrate lithium-rich layered surfaces with a framework matching the host's, but with nickel atoms regularly arranged between layers.

## Graphical abstract

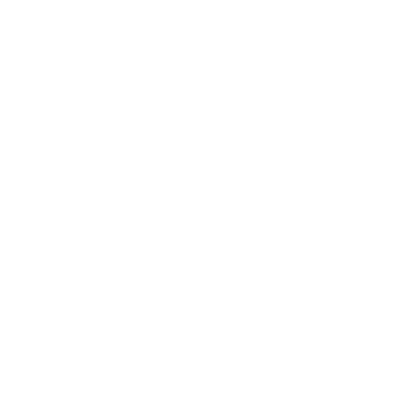

The limited specific capacity of cathode materials is one of the main obstacles to increasing the energy densities of current lithium-ion batteries^1^,^2^,^3^,^4^,^5^,^6^,^7^. In this regard, Li-rich layered oxides with the chemical formula of *x*Li_2_MnO_3_·(1−*x*)Li*M*O_2_ (*M*=3*d* and/or 4*d* transition metal; TM)^8^,^9^,^10^,^11^,^12^ have attracted significant attention because their specific capacities usually exceed 200 mAh g^−1^ at high operating voltages over 3.5 V versus Li^+^/Li, in contrast with their conventional layered counterparts, that is, LiCoO_2_ (∼145 mAh g^−1^) and LiCo_1/3_Mn_1/3_Ni_1/3_O_2_ (∼165 mAh g^−1^)^13^. Despite the resulting high energy density, most of these materials suffer from voltage drop and capacity fading during cycling, limiting their use in practical cells.

To achieve a high specific capacity, Li-rich layered oxides must be activated in the first charge involving Li ion extraction and oxygen evolution. During this process, TMs are liable to migrating to the neighbouring Li slabs (Fig. 1a)^8^,^14^,^15^,^16^,^17^, driving a phase transition of the overall crystal framework to spinel and/or rocksalt structures. This spontaneous phase transition continues with cycling and causes a drop in the operation voltage and specific capacity^18^,^19^,^20^, which undermines the original advantage of the Li-rich layered phase.

A number of approaches have been introduced to mitigate this unwanted phase transition. Surface modification has been one of the primary remedies because the given phase transition typically begins from the surface of the particle^21^,^22^,^23^, and well-designed surface structures can suppress this transition process from the initial stage^24^,^25^. In this direction, various surface-coating materials, namely, metal oxides^26^,^27^, metal phosphates^28^, spinel-type materials^29^,^30^ and olivine-type materials^20^, have been reported (Fig. 1b). Despite the substantial contributions of these coating materials towards cycle life enhancement, it is still desirable to find a new surface structure with further improved properties. To this end, one can raise a fundamental question: what would be the ideal surface structure for Li-rich layered oxides? In answering this question, a good surface structure would have a homogenous crystal structure at the atomic level with the host Li-rich layered phase so that the surface structure can be uniformly formed over the entire particle surface during synthesis^31^,^32^ and can also remain integrated with the host phase during repeated charge–discharge cycles. In this line, the previous surface modifications with different crystal structures have lattice mismatches and are therefore expected to suffer from disintegration during prolonged cycling, as well as non-uniform integration in the pristine state. More critically, a good surface structure of Li-rich layered oxides should be able to suppress the aforementioned transitions. A good surface structure is also desired to be electrochemically active with decent ionic/electronic conductivity so that it does not sacrifice the high specific capacity of the host Li-rich material.

In this work, we develop a surface structure for Li-rich layered cathodes. The surface structure addresses the issues of Li-rich layered oxides and thus improves their structural stability during repeated charge–discharge cycles. In particular, in an effort to minimize the difference in interfacial free energy (thus, formation energy)^33^,^34^ with the host material (Li-rich layered phase) and preserve the high electrochemical properties at the surface, Li_2_MnO_3_ with a consistent crystal framework to the host is adopted. Moreover, the Li_2_MnO_3_ surface phase is further modified in such a way that nickel (Ni) is regularly arranged between the face-to-face Li sites in the superlattice manganese (Mn) layers of Li_2_MnO_3_ (Fig. 1c). The presence of Ni in the 3 nm-thick surface layer effectively suppresses phase transition to spinel and/or rocksalt structures, leading to improved cycling and rate performance.

## Results

### Material synthesis and structure characterization

**Figure 2:**
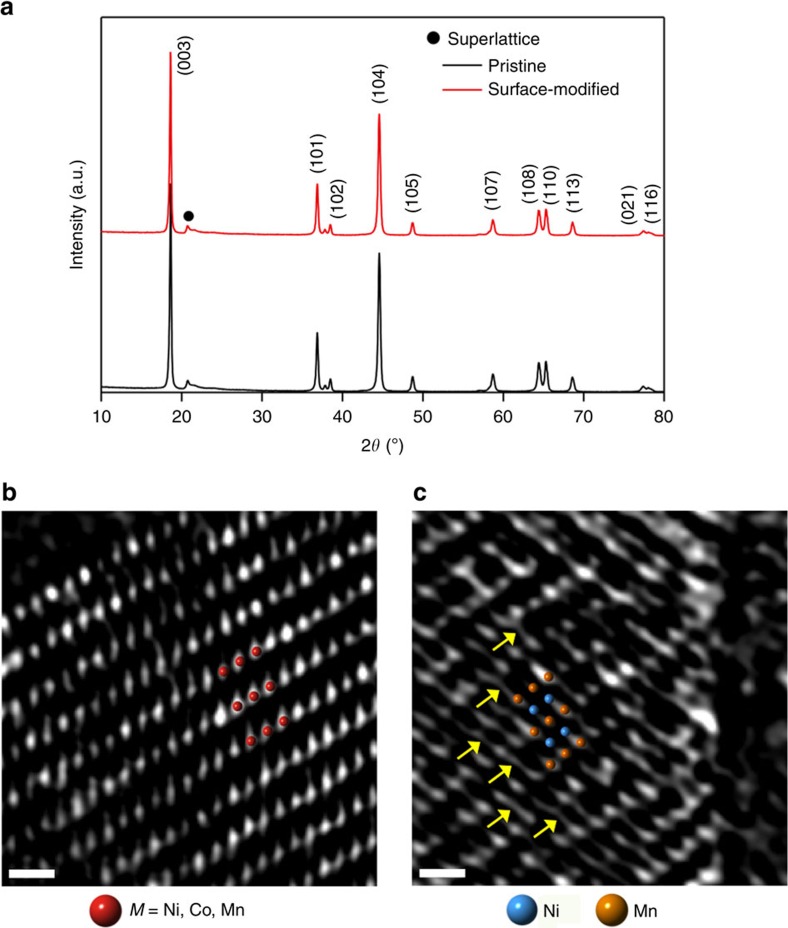
Crystal structures before and after surface modification. (**a**) X-ray diffraction patterns of the pristine electrode and the surface-modified electrode with 5 wt% Li_2_MnO_3_. (**b**,**c**) HAADF-STEM images of (**b**) the pristine electrode and (**c**) the surface-modified electrode with 5 wt% Li_2_MnO_3_. **b**,**c** were obtained along the rhombohedral [100] and monoclinic [010] directions, respectively. The red, blue and orange circles represent TM (*M*=Ni, Co and Mn), Ni and Mn, respectively. Scale bars, 5 Å.

In our experiment, Li_2_MnO_3_ was first coated on a Li-rich layered oxide, 0.5Li_2_MnO_3_–0.5LiNi_0.44_Mn_0.32_Co_0.24_O_2_. The modification of the surface structure to position Ni in the Li layers was achieved by inter-diffusion of Ni from the host during a heat treatment in the coating process. See the Methods section for details and Supplementary Note 1 for additional description of the bulk and surface materials used, including their crystal structures (Supplementary Fig. 1), X-ray diffraction patterns (Supplementary Fig. 2a), and transmission electron microscopy (TEM; Supplementary Fig. 2b,c) characterization. The diffraction patterns were indexed based on a hexagonal unit cell with the space group 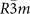 (No. 166)^17^. With increasing Li_2_MnO_3_ content, the normalized intensity of the peak at 20.83°, which is reflective of the superlattice, increases gradually (Fig. 2a, Supplementary Fig. 3 and Supplementary Table 1). This superlattice is associated with the repeated 2:1 atomic arrangement between TM and Li in the TM layers^10^. As neither impurity phases nor significant peak shifts were detected for the modified sample, the increased intensity of the superlattice peak indicates the formation of a Li_2_MnO_3_-like phase on the surface without perturbing the host framework. The lattice parameters before and after the surface modification were calculated to be *a*_hex_=2.802(3) Å, *c*_hex_=14.174(1) Å and *a*_hex_=2.801(1) Å, *c*_hex_=14.171(7)Å, respectively, verifying that the surface phase was structurally well linked to the host framework. The surface layers were coated over all of the host particles in a uniform fashion with well-controlled thickness around 3 nm according to our TEM characterization with multiple particles (Supplementary Fig. 4).

High-angle annular dark field-scanning TEM (HAADF-STEM) images of both the pristine and surface-modified electrodes display typical layered atomic columns (red circles in Fig. 2b and orange circles in Fig. 2c and Supplementary Fig. 5, respectively) based on their *Z*-contrast^35^. Interestingly, in the case of the surface-modified electrode, additional atomic columns (blue circles and yellow arrows) were observed in the Li layers of the surface-modified region. Since light elements, such as Li and O, are invisible in HAADF imaging^36^,^37^, the bright contrasts between the TM layers indicate the existence of heavy TMs, as the given modified material consists solely of TMs, Li and O. In addition to STEM characterization, energy-dispersive X-ray spectroscopy (EDX) analyses showed that there was a high Ni content across the surface region of the modified electrode (Supplementary Fig. 6). Thus, these combined analyses suggest the diffusion of Ni atoms from the host to the surface during the heat treatment. The distance (∼3 nm) from the surface to the far edge, where the interlayer Ni begins to be found, agrees well with the thickness of the Ni layer in the EDX analysis (Supplementary Fig. 6) and the coating thickness in the low-magnification TEM images (Supplementary Fig. 4). Given the fact that at this outermost region the primary phase is Li_2_MnO_3_ and a high Ni content is found, it can be concluded that the observed orange and blue atomic columns in the TM and Li layers are Mn and Ni, respectively. The well-known high diffusivity of Ni during heat treatment^38^,^39^ is also in keeping with Ni diffusion from the host region. The atomic resolution STEM images exhibited weaker contrast of Ni atoms compared with that of Mn atoms (Fig. 2c). Since the HAADF mode provides the contrast in proportion to the atomic density and the atomic number, the weaker contrast of the Ni atoms indicates their lower occupancy. On the other hand, the crystallinity of the modified surface at the pristine state was relatively lower, and therefore the superlattice arrangement between the Mn and Li atoms in the TM layers was difficult to observe in the atomic resolution. However, the given superlattice arrangement was clearly detected from the X-ray diffraction results (Fig. 2a, Supplementary Fig. 3 and Supplementary Table 1). According to X-ray photoelectron spectroscopy spectra (Supplementary Fig. 7), Mn^3+^ was detected, indicating that some Mn in Li_2_MnO_3_ was reduced in the modified surface because of the Ni migration that supplies additional positive charge. See Supplementary Note 2 for detailed description on the Ni diffusion. With this substantial presence of Ni, the blue columns are unlikely to represent Mn because the structure would have then undergone serious Mn migration to the Li slabs, and this TM mixing would have resulted in a phase transition to a spinel-like structure^40^,^41^. Because of the low Co content in the EDX profile (Supplementary Fig. 6), the blue columns are also unlikely to represent Co. Additional structural description of the surface-modified electrode is provided in Supplementary Note 2.

### Electrochemical properties

**Figure 3:**
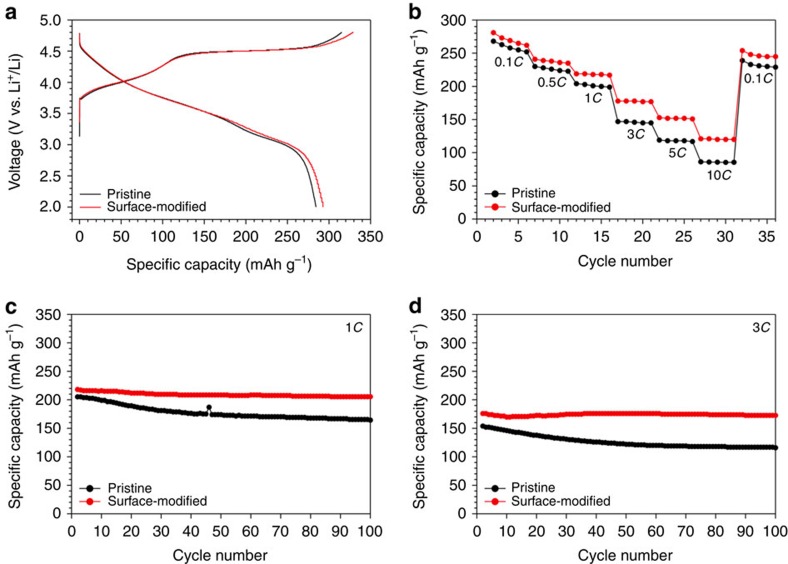
Electrochemical performance before and after surface modification. (**a**), The first charge–discharge voltage profiles of the pristine electrode and the surface-modified electrode with 5 wt% Li_2_MnO_3_ in the range of 2.0–4.8 V at 0.1*C* (25 mA g^−1^). (**b**) Comparative rate capabilities of both electrodes measured at various *C*-rates after the first activating cycle at 0.1*C* (25 mA g^−1^). (**c**,**d**) Cycling performance of both electrodes at (**c**) 1*C* (250 mA g^−1^) and (**d**) 3*C* (750 mA g^−1^) after the first activating cycle at 0.1*C* (25 mA g^−1^).

The surface-modified electrode exhibited good electrochemical performance in various aspects, such as specific capacity, rate capability and cycle life. Detailed cell preparation and measurement conditions are described in the Methods section. From the testing of various contents of Li_2_MnO_3_ (Supplementary Fig. 8), 5 wt% was chosen as a main surface-modified electrode in the current investigation. When galvanostatically tested in the voltage range of 2.0–4.8 V versus Li^+^/Li at 0.1*C* (25 mA g^−1^), the surface-modified electrode exhibited higher first charging and discharging capacities than the pristine electrode (Fig. 3a). In the first charge, the surface-modified and pristine electrodes delivered 329.4 and 315.0 mAh g^−1^, respectively, whereas in the first discharge, they showed 292.7 and 284.2 mAh g^−1^, respectively. The larger charge capacity of the surface-modified electrode is ascribed to its higher content of a Li_2_MnO_3_-like phase with a high specific capacity. The magnified discharge profile (Supplementary Fig. 9) of the surface-modified electrode exhibited weakened voltage fading around 3.0 V, which implies that the suppressed phase transition contributes to the increased discharge capacity. By contrast, most of the previous approaches involving other substitutional elements that are indiscreetly distributed in the Li interlayer (often referred to pillar atoms) impair Li ion migration and consequently sacrifice the intrinsic electrochemical performance of the host Li-rich material^42^,^43^,^44^.

The surface-modified electrode also displayed improved rate capability (Fig. 3b, and Supplementary Figs 8 and 10). After the activation in the first cycle, as the *C*-rate increased by 5, 10, 30, 50 and 100 times from 0.1*C* (1*C*=250 mA g^−1^), the surface-modified electrode retained 85.8%, 77.9%, 63.3%, 55.4% and 43.1% of the capacity (281.2 mAh g^−1^) in the second cycle, respectively. By contrast, with the same *C*-rate variations, the pristine electrode retained 85.8%, 76.1%, 54.8%, 44.4% and 32.0%, even though its capacity (268.4 mAh g^−1^) in the second cycle was lower.

More critically, the surface-modified electrode showed better cycling performance than the pristine electrode. When measured at 0.1*C* (25 mA g^−1^), the discharge capacity of the surface-modified electrode was 292.3 mAh g^−1^ in the second cycle, and this value dropped only to 259.7 mAh g^−1^ after 45 cycles, corresponding to an 88.8% capacity retention (Supplementary Fig. 11). After the same number of cycles, however, the pristine electrode retained only 80.9% with respect to the capacity (279.7 mAh g^−1^) in the second cycle. When measured at higher rates of 1*C* (250 mA g^−1^) and 3*C* (750 mA g^−1^), 94.1 and 98.2% of the capacities (218.3 and 176.0 mAh g^−1^) in the second cycle were preserved for the surface-modified electrode after 100 cycles (Fig. 3c,d). These values are in contrast with those (80.1 and 75.3%) of the pristine counterpart. Remarkably, unlike the pristine electrode, the discharge capacity of the surface-modified electrode became saturated even after several cycles. These capacity retentions are quite noticeable, as they are better than those of other nano-structured^45^,^46^, cation-doped^47^,^48^,^49^ and surface-coated^20^,^26^,^27^,^28^,^29^,^30^,^50^ Li-rich layered oxide electrodes reported to date. Consistent with the first cycle, the improved capacity retentions over the prolonged cycles are attributed to the suppressed phase transition, as verified by long-term cycling (Supplementary Figs 12 and 13) and differential capacity results (Supplementary Fig. 14). More electrochemical results are provided in Supplementary Note 3.

### Reaction mechanism

**Figure 4:**
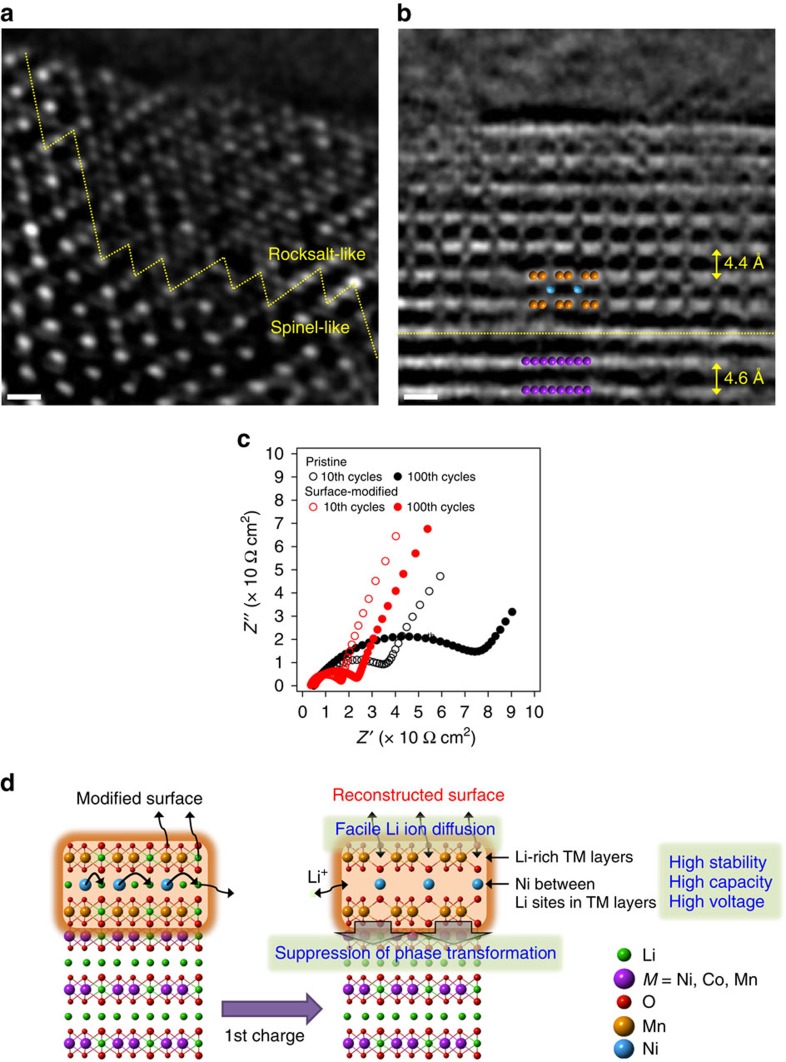
Effect of atomic-level surface modification. (**a**,**b**) HAADF-STEM images of (**a**) the pristine electrode and (**b**) the surface-modified electrode with 5 wt% Li_2_MnO_3_ after the first charge. **a**,**b** were obtained along the rhombohedral [100] (=cubic spinel [110]) and monoclinic [100] directions, respectively. (**c**) Nyquist plots of both electrodes after the tenth and hundredth cycles. (**d**) Graphical illustration of the effect of the surface modification. The green, purple, red, orange and blue circles represent Li, TM (*M*=Ni, Co and Mn), O, Mn and Ni, respectively. Scale bars, 5 Å.

The origin of the improved electrochemical stability by the surface modification was revealed in detail by HAADF-STEM analysis. The HAADF-STEM image of the pristine electrode after the first charge displays rocksalt-like and spinel-like phases sequentially from the surface (Fig. 4a), indicating that spontaneous transition to these phases progresses quite a bit even in the early period of cycling. By contrast, in the same charged state, the surface region of the modified electrode kept the original layered structure (Fig. 4b). In particular, the image of the outermost region (the area above the yellow dotted line) provides the atomic-scale information on its crystal structure on charge. As opposed to the pristine electrode, once again, the surface region preserves the layered framework all the way to the edge, indicating its structural robustness. In many regions, the repeated 2:1 superlattice arrangement of the Mn and Li atoms in the TM layers was clearly detected; one blank spot reflective of the original Li sites (now emptied on charge) was observed after every two orange circles. Also, similar to the state before cycling, Ni atoms were observed in the Li layers, and a majority of the Ni atoms are located right next to the face-to-face Li sites located in the TM layers (Fig. 4b). On the whole, it appears that the atoms in the surface region are ordered in the superlattice configuration, presumably because, on Li ion extraction, the Ni atoms in the Li layers migrate and settle at the positions next to the face-to-face Li sites. This atomic rearrangement reflects the enhanced crystallinity of the surface layer during charge, although analyses to identify detailed mechanism is underway. This trend was confirmed from multiple spots along the surface and also from multiple particles (Supplementary Fig. 15).

By the assistance of this robust layered surface structure incorporating Ni atoms specifically located at the 2*c* sites, the inner region (the area below the yellow dotted line) preserves the original layered framework (purple circle) with no cation mixing. In Li-rich layered oxides, it is known^22^,^51^ that Li extraction is accompanied with the oxidation of oxygen atoms to O^(2-δ)−^, which can lead to the oxygen loss. The oxidation of oxygen perturbs its stability via delocalization of the bonding electrons^52^,^53^. It was reported^54^ that the structural stability is deteriorated more significantly by the 4*i* oxygen vacancy than the 8*j* oxygen vacancy. Once the 4*i* oxygen atoms are destabilized, the TM migration through the adjacent tetrahedral sites would be accelerated. In this regard, the improved structural stability is credited to the Ni coordination (2*c* sites) to the adjacent TM layers because the Ni atoms form strong bonding with the adjacent oxygen atoms (4*i* sites) and suppress the TM migration through the adjacent tetrahedral sites based on high electrostatic repulsion between the TMs and Ni atoms. The decreased interlayer distance (4.4 Å) in the modified surface compared with that (4.6 Å) in the host directly reflects the strong bonding between TMs and oxygen atoms in the surface region (Fig. 4b). Thus, it can be concluded that the robust superlattice surface structure enabled by the well-defined Ni coordination (2*c* sites) plays a crucial role in inhibiting the structural changes that would otherwise propagate from the surface. It is well known^8^,^14^,^15^,^16^,^17^ that in Li-rich layered oxides cation mixing is a spontaneous process at the very beginning of the first charging, and even the migration of a small portion of TMs can initiate structural disordering sufficient for eventual phase transition to spinel and/or rocksalt phases. In this sense, the observed layered surface structure with high atomic regularity is remarkable. The structural stability of the surface-modified electrode was also verified by *ex situ* X-ray diffraction analysis (Supplementary Fig. 16).

Electrochemical impedance spectroscopy (EIS) analyses also reveal the more stable electrode/electrolyte interface of the surface-modified electrode for 100 cycles compared with the pristine electrode (Fig. 4c and Supplementary Fig. 17); the semi-circle of the surface-modified electrode in the EIS curve stays smaller and more steady throughout cycling. Detailed EIS results are summarized in Supplementary Table 2. The lower interfacial resistance of the surface-modified electrode can be explained by its superlattice layered structure with facile Li ion diffusion (Supplementary Fig. 18 and Supplementary Table 3), as well as mitigated electrolyte side reactions due to the stable electrode surface. In the same line, the excessive and indiscreet Ni arrangement in the Li interlayer could hinder the Li ion diffusion^42^, sacrificing the electrochemical properties of the host material. The effect of the modified surface on the structural and interfacial stability is graphically summarized in Fig. 4d and is further described in Supplementary Note 4.

## Discussion

We have demonstrated a robust surface structure for Li-rich layered oxide cathodes. The Li-rich layered framework of the modified surface with atomic-level structural consistency with the host phase allows the surface region to remain seamlessly connected during battery operation. Moreover, in this modified surface, Ni atoms are regularly positioned between the TM layers and maintain the overall Li-rich layered framework. To the best of our knowledge, the prepared Li-rich surface structure, in which TMs are located specifically between the face-to-face Li sites in the TM layers, has never been reported previously. This unique atomic coordination significantly suppresses a detrimental transition to spinel and/or rocksalt phases by mitigating TM mixing, a main trigger step of the phase transition.

The present surface structure is also clearly distinct from a number of surface-coating approaches to date because the majority of the reported surface materials adopted different structures from the host materials and therefore impaired the specific capacities and electronic and/or ionic conductivities of the host materials. Although the Ni atoms at the 2*c* sites do not block the channels for Li ion diffusion, the high Ni content would decrease the Li content, which could affect the Li diffusivity and the reversible capacity.

Complementing the structural stability and electrochemical performance, the surface structure achieved in this study was created through simple dip-dry and heat-treatment processes, which neither perturb the host framework nor generate any impurities. Taking the present work as the first step, detailed properties (thickness, Ni content and so on) related to the surface structure can be further optimized by tuning the surface-to-bulk ratio and heat treatment condition. Although a surface structure based on NiO rocksalt was reported to stabilize Ni-rich layered cathodes^42^, the given approach is not appropriate to form Li-rich surface phases as in the present case. Highly important is the fact that this study provides a general design principle on how to build the surface structure, targeting a variety of battery electrode materials that suffer from structural collapse starting heavily from their surfaces: creating a surface structure with the same crystal structure as the host phase, followed by a heat treatment to stimulate atomic interaction with the host structure. During the interaction, the surface structure would modify itself and transform to a more stable phase, as the present material demonstrated the inter-diffusion of highly diffusive Ni from the host phase to the specific crystal sites that can play a critical role in keeping the framework during Li ion extraction.

## Methods

### Synthesis

Commercial 0.5Li_2_MnO_3_–0.5LiNi_0.44_Mn_0.32_Co_0.24_O_2_ (BASF SE)^55^,^56^ was used for both the pristine and surface-modified electrodes. First, 1 g of 0.5Li_2_MnO_3_–0.5LiNi_0.44_Mn_0.32_Co_0.24_O_2_ was dispersed in 10 ml of anhydrous ethanol by ultrasonic stirring for 12 h. All of the chemicals were purchased from Sigma-Aldrich and used without any purification. Lithium acetate (LiCOOCH_3_·2H_2_O) and manganese acetate (Mn(COOCH_3_)_2_·4H_2_O) were added in a 2:1 molar ratio to the host material suspension, and this suspension was vigorously stirred for 12 h. The suspension was then heated at 70 °C until its solvent evaporated. The remaining powder was calcined at 600 °C for 6 h to obtain the final surface-modified sample. The Li_2_MnO_3_ content was controlled to be 1, 3, 5, 10 and 20 wt%.

### Characterization

The crystal structure was investigated by X-ray diffraction (Rigaku) analysis in the 2*θ* range of 10°–80° with a scan step of 0.02° and an acquisition time of 2 s for each step. The specimens were sealed in a homemade holder inside an Ar-filled glove box to prevent hydration from moisture in air. Morphology and elemental analyses were carried out by TEM (JEM-ARM200F, JEOL) characterization at an acceleration beam voltage of 300 kV and its attached EDX (Quantax 400, Bruker), respectively. HAADF-STEM characterization was performed using a JEM-ARM200F (JEOL) equipped with a cold field-emission gun and double spherical aberration correctors, operated at an acceleration voltage of 200 kV. The probe beam had a convergent semi-angle of 23 mrad. The inner-outer semi-angle of the HAADF detector was 90–370 mrad. The electron probe current was 11 pA. The dwell time per pixel was 38 μs. The size of each pixel was 16.5 × 16.5 pm^2^. The oxidation states of TMs were examined using X-ray photoelectron spectroscopy (Thermo Scientific Sigma Probe). The impedance was measured using a frequency response analyser (VSP multi potentiostat, BioLogic) over the frequency range of 0.01 Hz–1 MHz with an amplitude of 10 mV.

### Electrochemical test

For electrode fabrication, a slurry consisting of 80 wt% of active material, 10 wt% of super P and 10 wt% of poly(vinylidene fluoride) was first prepared. The slurry was then cast onto aluminum foil using the doctor blade technique, followed by drying at 70 °C for 12 h in a vacuum oven. The mass loading of the active material in each electrode was ∼4.0 mg cm^−2^. Li foil was used as the counter and reference electrode. The electrolyte solution was 1 M lithium hexafluorophosphate (LiPF_6_) dissolved in an ethylene carbonate/dimethyl carbonate mixture (1:1 v/v). Polypropylene membranes (2,400, Celgard) were used as separators. These cell components were assembled in the form of CR2032 coin cells in an argon-filled glove box. All of the cells were aged for 6 h before any electrochemical processes to ensure sufficient soaking of the electrolyte into the separator and electrode. The cells were cycled in the voltage range of 2.0–4.8 V (versus Li^+^/Li) at 25 °C using a battery tester (WBCS3000L, Wonatech). The *C*-rate in this study is defined based on 1*C*=250 mA g^−1^. For the calculation of all of the gravimetric capacities/currents, the mass of the active material only was taken into account.

### Data availability

All relevant data are available from the authors on request.

## Additional information

**How to cite this article:** Kim, S. *et al*. A stable lithium-rich surface structure for lithium-rich layered cathode materials. *Nat. Commun.* **7,** 13598 doi: 10.1038/ncomms13598 (2016).

**Publisher's note**: Springer Nature remains neutral with regard to jurisdictional claims in published maps and institutional affiliations.

## Supplementary Material

Supplementary Information Supplementary Figures 1 - 18, Supplementary Tables 1- 3, Supplementary Notes 1 - 4 and Supplementary References

## Figures and Tables

**Figure 1:**
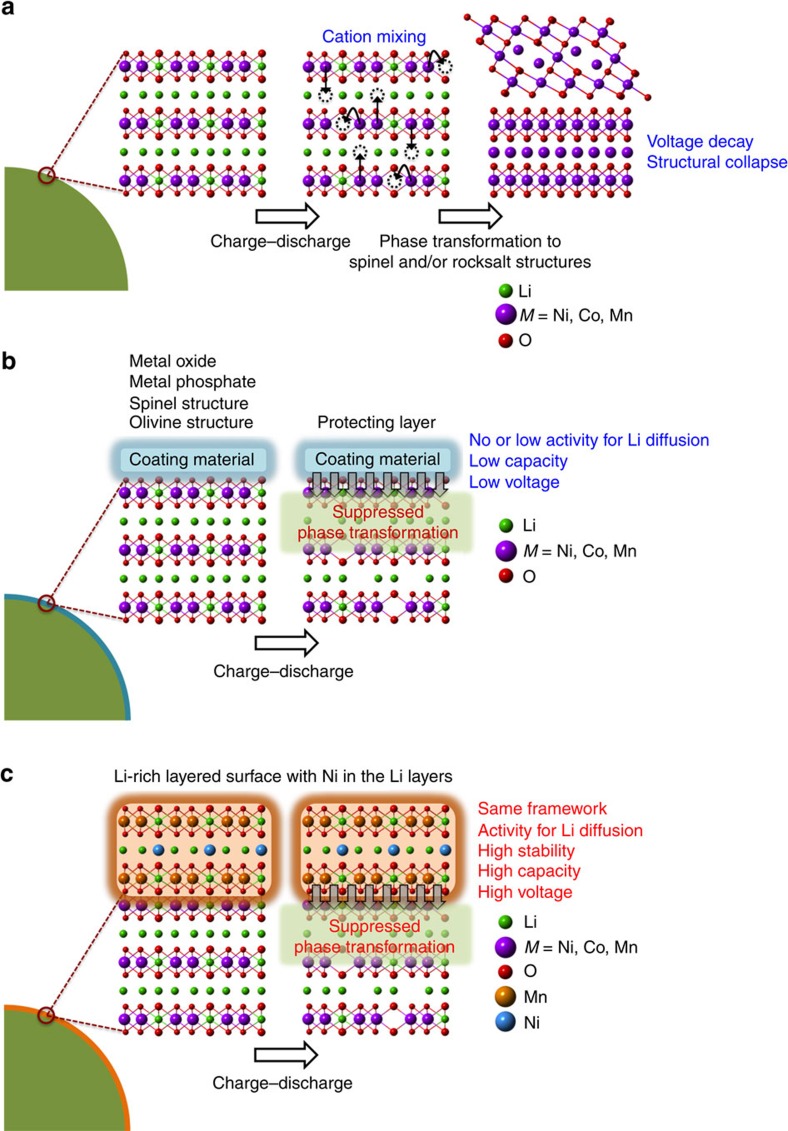
Crystal structures near the surfaces of Li-rich layered oxides. (**a**) Without any surface modifications. (**b**) With conventional surface coating. (**c**) With surface modification in which Ni is regularly positioned between the face-to-face Li sites of the superlattice Mn layers in the Li-rich layered surface structure.
