# Supplementary material for: A stable lithium-rich surface structure for lithium-rich layered cathode materials
Source: Nat Commun. 2016 Nov 25;7:13598. doi: 10.1038/ncomms13598 (PMC5133657; doi:10.1038/ncomms13598)
Supplement: Supplementary Information — Supplementary Figures 1 - 18, Supplementary Tables 1- 3, Supplementary Notes 1 - 4 and Supplementary References [file ncomms13598-s1.pdf]

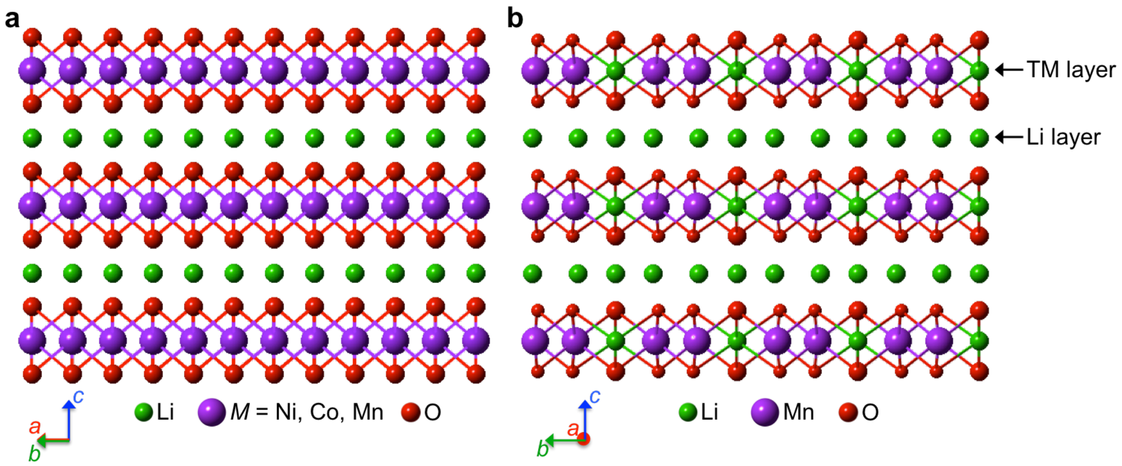

Supplementary Figure 1. Crystal structures of conventional layered and Li-rich layered manganese oxides. a, The crystal structure of rhombohedral LiMO$_2$ (M = Ni, Co, Mn) with the space group $R\overline{3}m$. **b,** The crystal structure of monoclinic Li_2MnO_3 with the space group $C2m$.

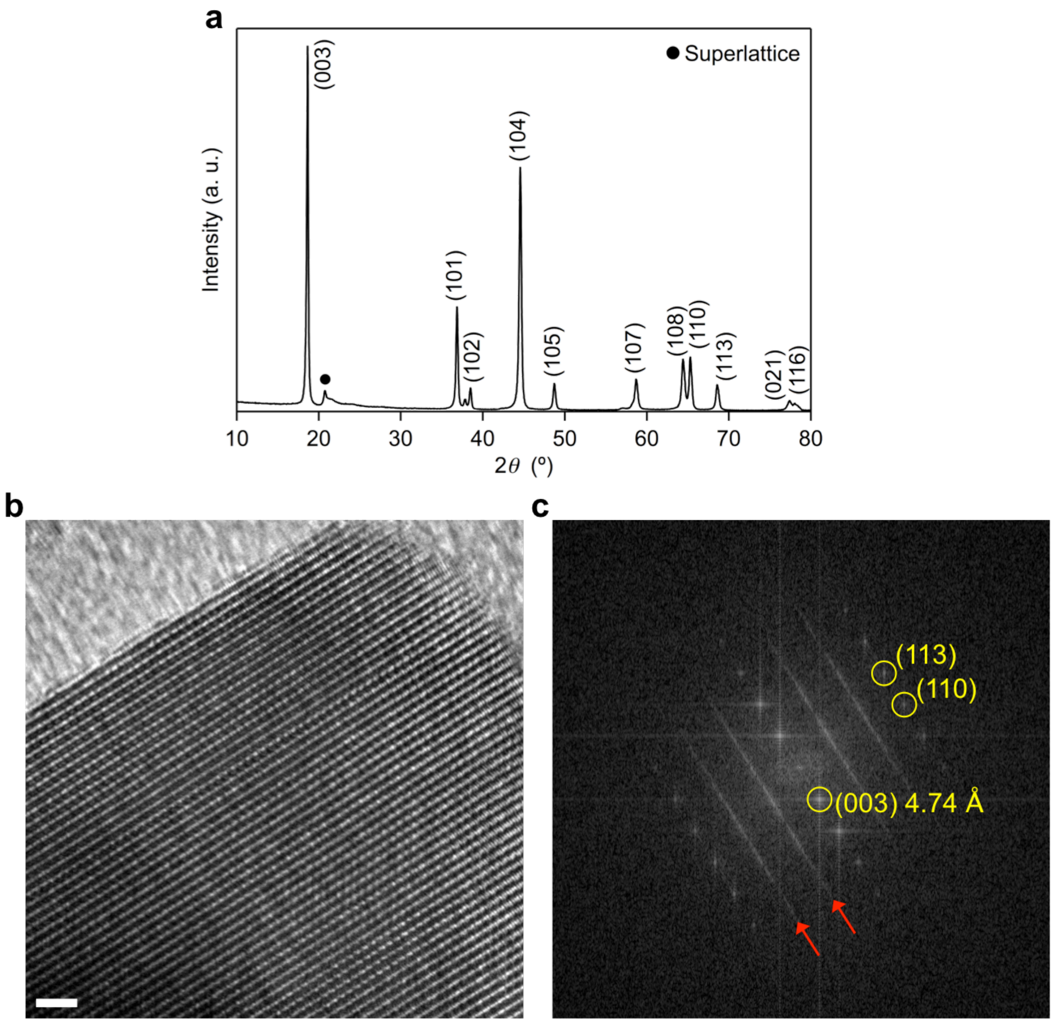

Supplementary Figure 2. Structure analysis of 0.5Li_2MnO_3-0.5$LiNi_{0.44}Mn_{0.32}Co_{0.24}O_2$. a, XRD pattern. **b,** TEM image. **c,** FFT pattern from b. Scale bar, 2 nm.

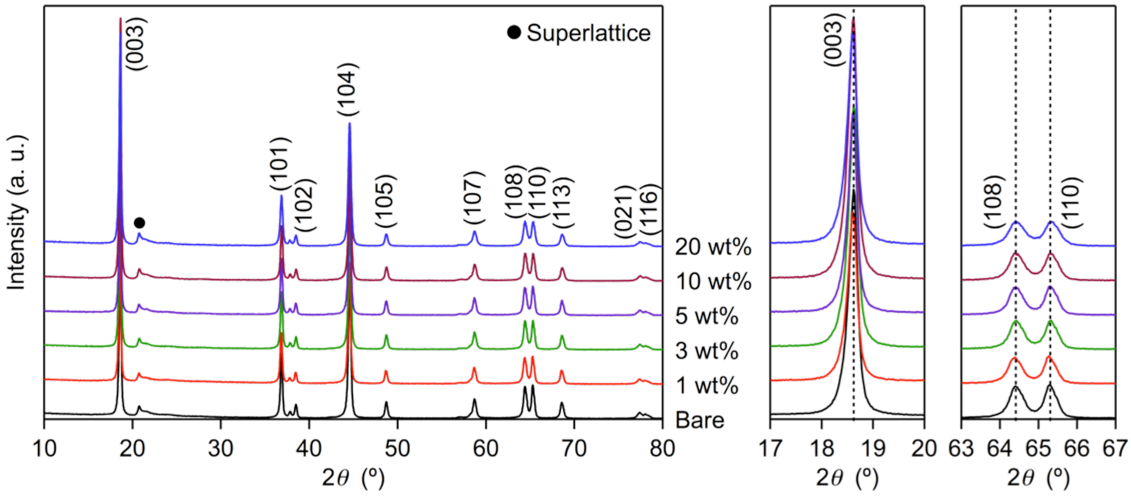

Supplementary Figure 3. Structure change after surface modification. The XRD patterns before and after surface modification involving various ratios of Li_2MnO_3.

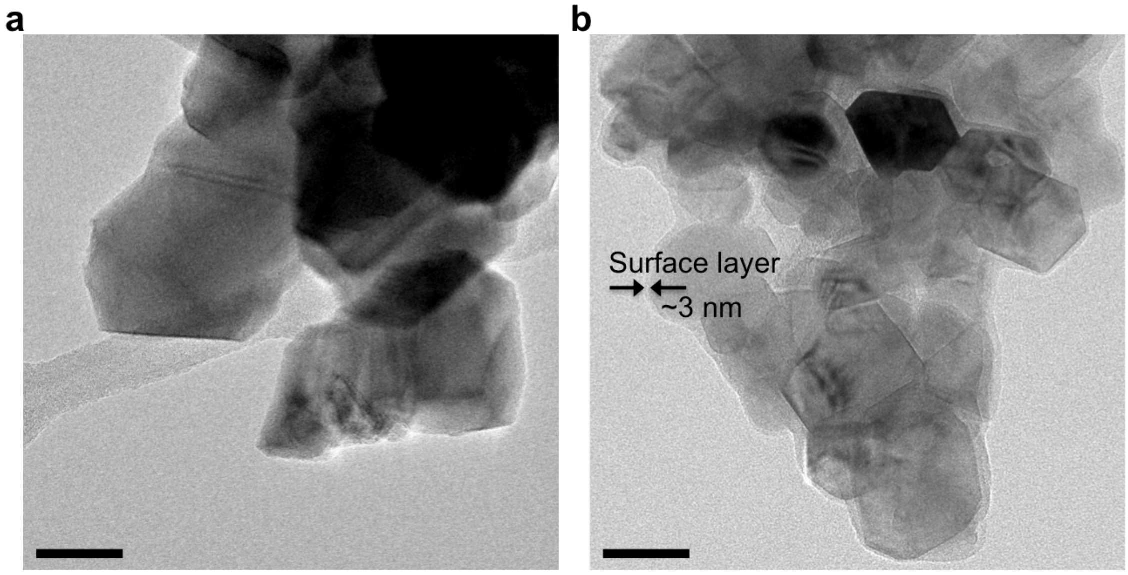

Supplementary Figure 4. Surface morphology change after surface modification. a, b, TEM images (**a**) before and (**b**) after surface modification using 5 wt% Li_2MnO_3. Scale bars, 50 nm.

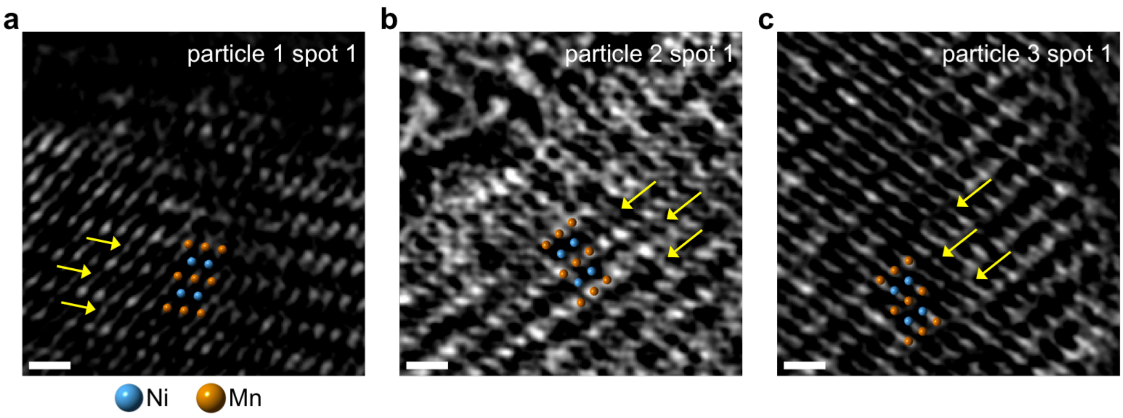

Supplementary Figure 5. Structure analyses of the surface-modified sample from multiple particles. a-c, HAADF-STEM images of the surface-modified electrode projected along the monoclinic [010] direction attained from different spots of three particles. Scale bars, 5 Å.

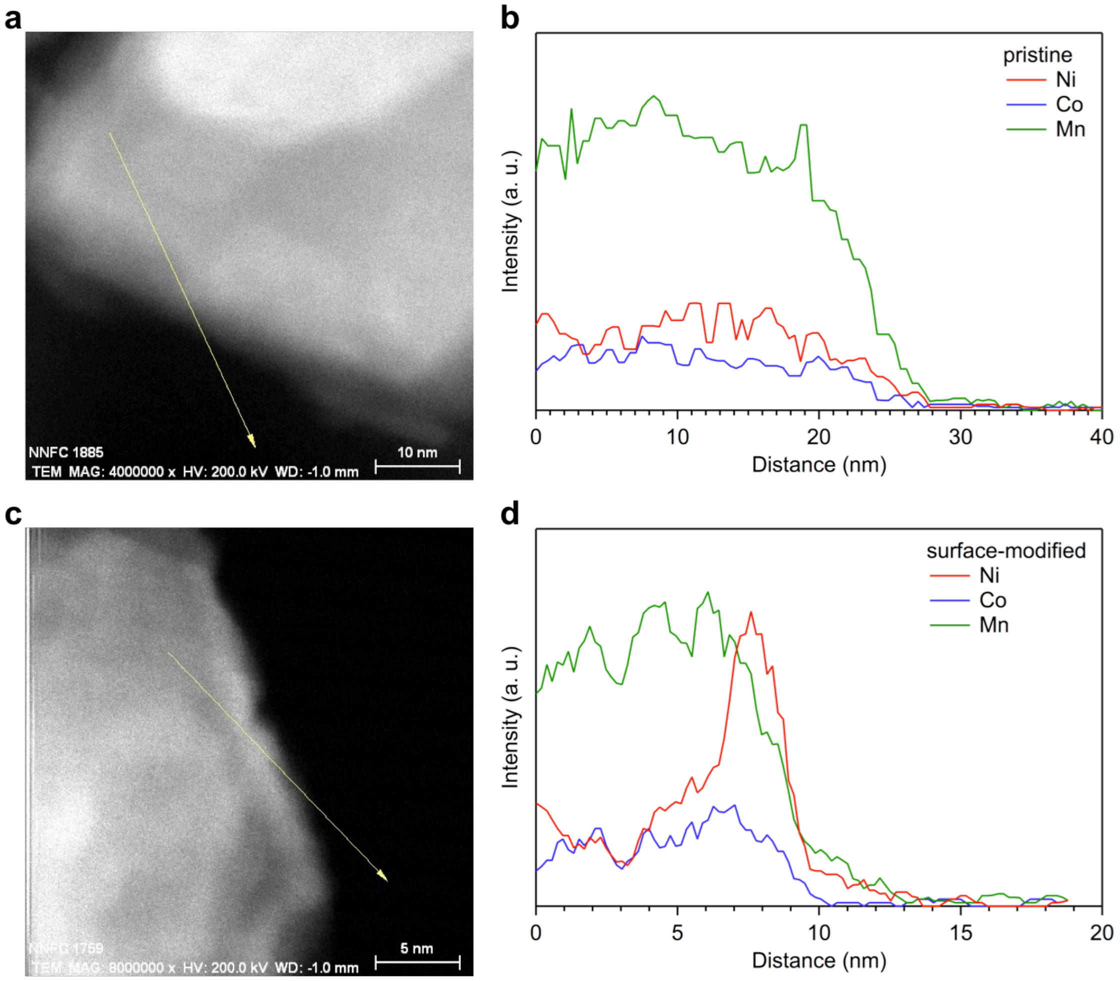

Supplementary Figure 6. Transition metal distributions before and after surface modification. a, b, (**a**) TEM image before surface modification and (**b**) its EDX line profiles with respect to three TMs along the line displayed in a. **c, d,** The same analyses for the surface-modified sample (5 wt% Li_2MnO_3).

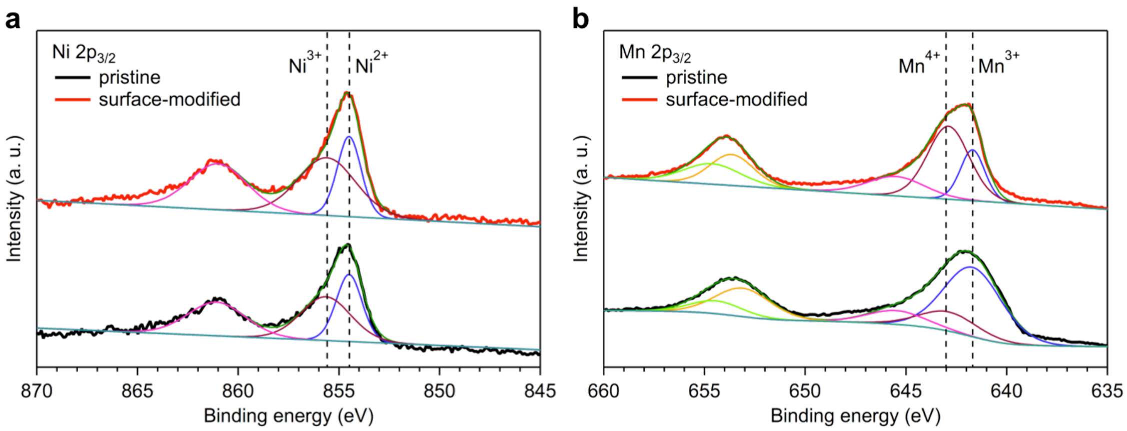

Supplementary Figure 7. The oxidation state of transition metals before and after surface modification. a, b, XPS spectra of (**a**) Ni $2p_{3/2}$ and (**b**) Mn $2p_{3/2}$ for the pristine electrode and the surface-modified electrode with 5 wt% Li_2MnO_3.

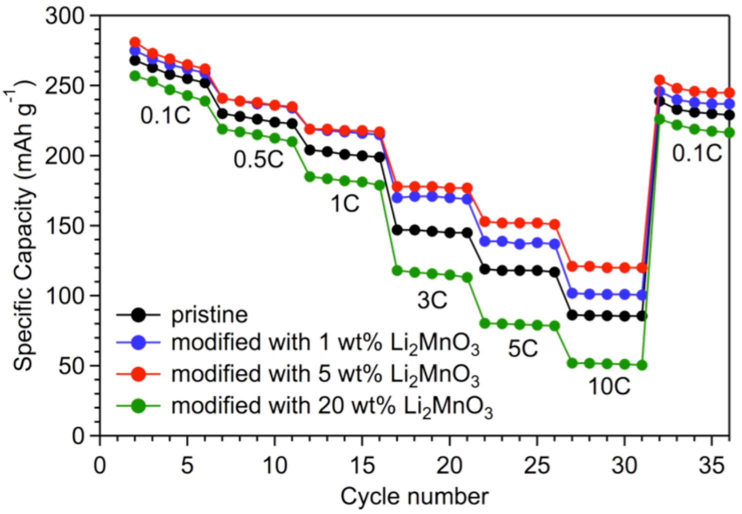

Supplementary Figure 8. Rate capability of the surface-modified electrodes containing various $LiMn_2O_3$ contents. The surface-modified electrodes possess 1 wt%, 5 wt%, and 20 wt% Li_2MnO_3. The measurements were carried out at 0.1C, 0.5C, 1C, 3C, 5C, 10C after the initial activation cycle.

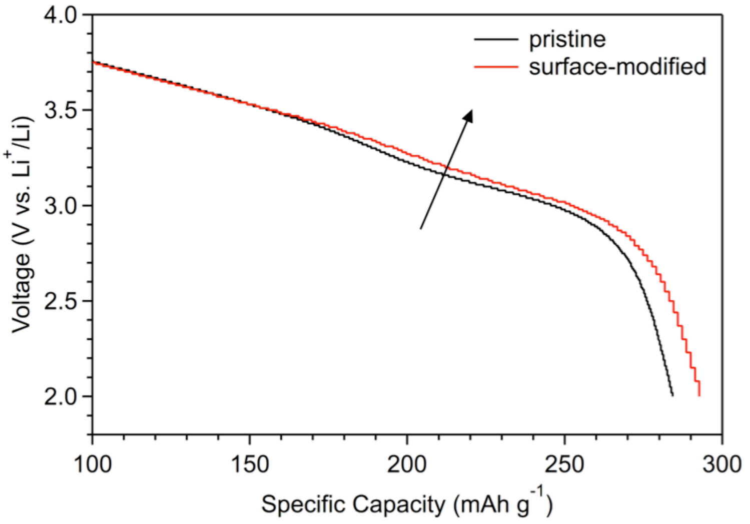

Supplementary Figure 9. Suppressed phase transformation after surface modification. Discharging profiles magnified from Fig 3a.

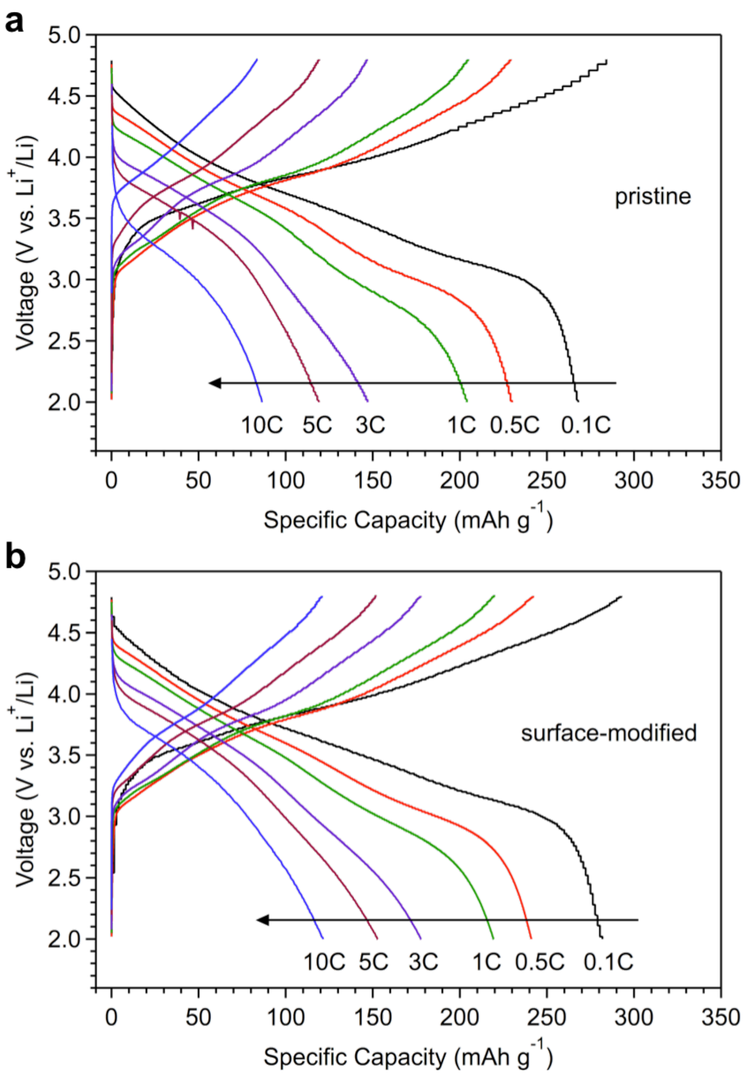

Supplementary Figure 10. Charge-discharge profiles at various C-rates. a, b, Charge-discharge profiles of (**a**) the pristine electrode and (**b**) the surface-modified electrode with 5 wt% Li_2MnO_3 at 0.1C, 0.5C, 1C, 3C, 5C, and 10C after the initial activation cycle.

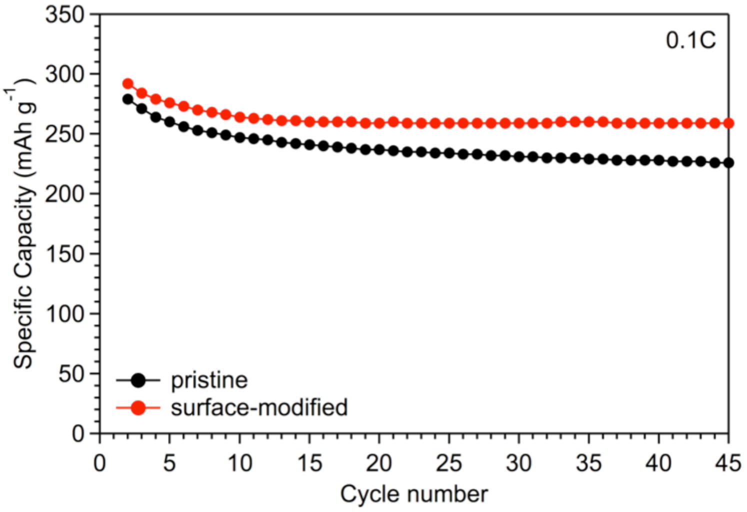

Supplementary Figure 11. Effect of surface modification on the cycling performance. Cycling performance of the pristine electrode and the surface-modified electrode with 5 wt% Li_2MnO_3 measured at 0.1C (25 mA g^{-1}) after the initial activation cycle.

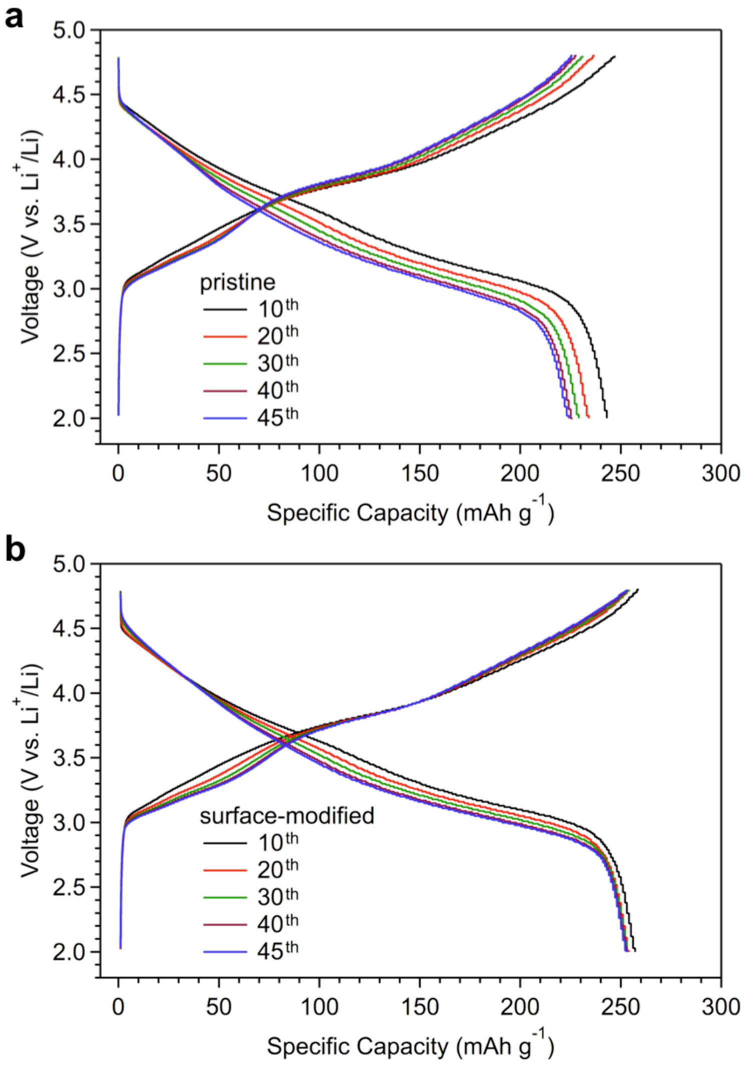

Supplementary Figure 12. Charge-discharge profiles for prolonged cycles. a, b, Charge-discharge profiles of (**a**) the pristine electrode and (**b**) the surface-modified electrode with 5 wt% Li_2MnO_3 during long-term cycling at 0.1C.

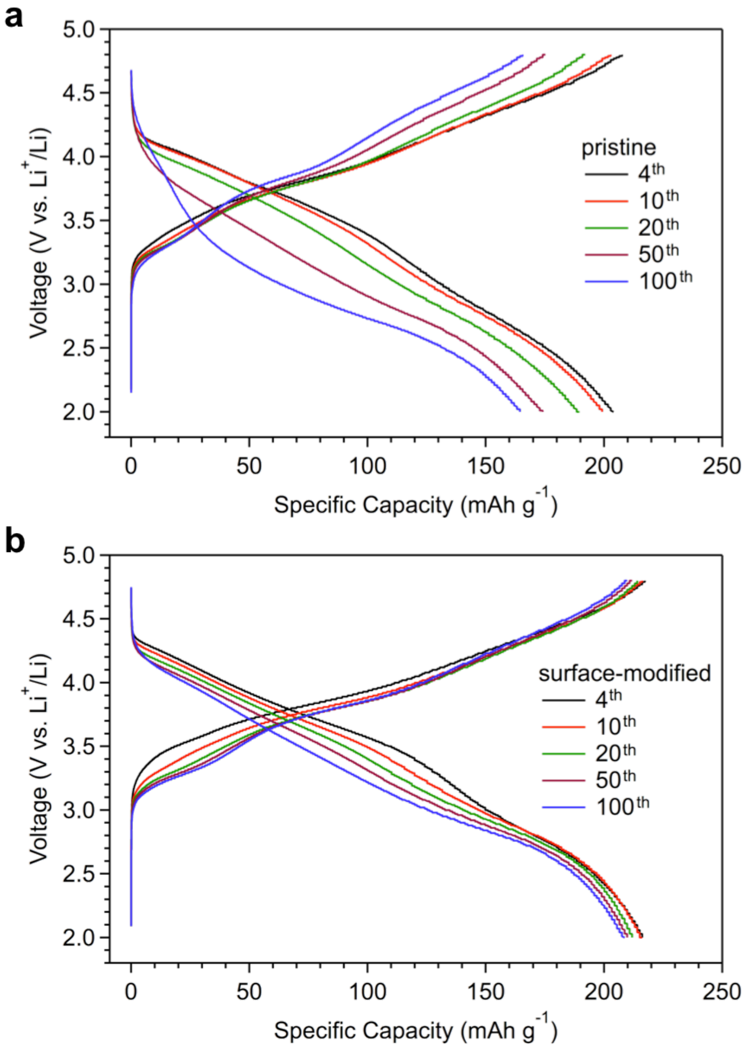

Supplementary Figure 13. Charge-discharge profiles for prolonged cycles. a, b, Charge-discharge profiles of (**a**) the pristine electrode and (**b**) the surface-modified electrode with 5 wt% Li_2MnO_3 during long-term cycling at 1C.

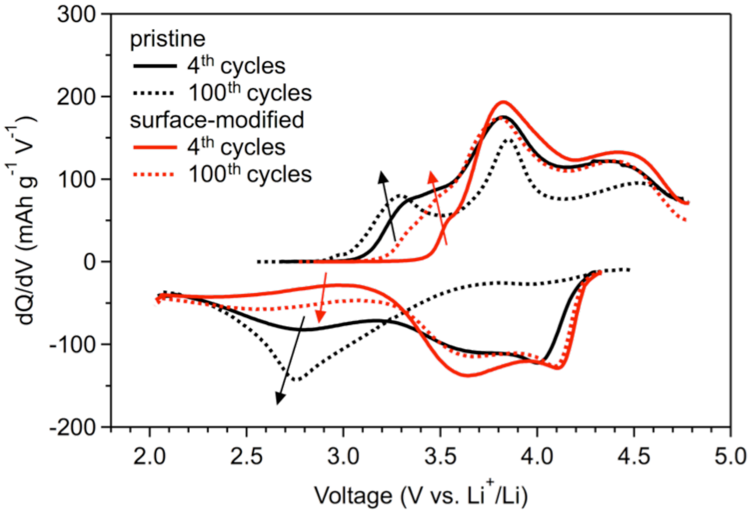

Supplementary Figure 14. Suppressed phase transition after surface modification. dQ/dV plots at the 4th and 100th cycles in Fig. 3c.

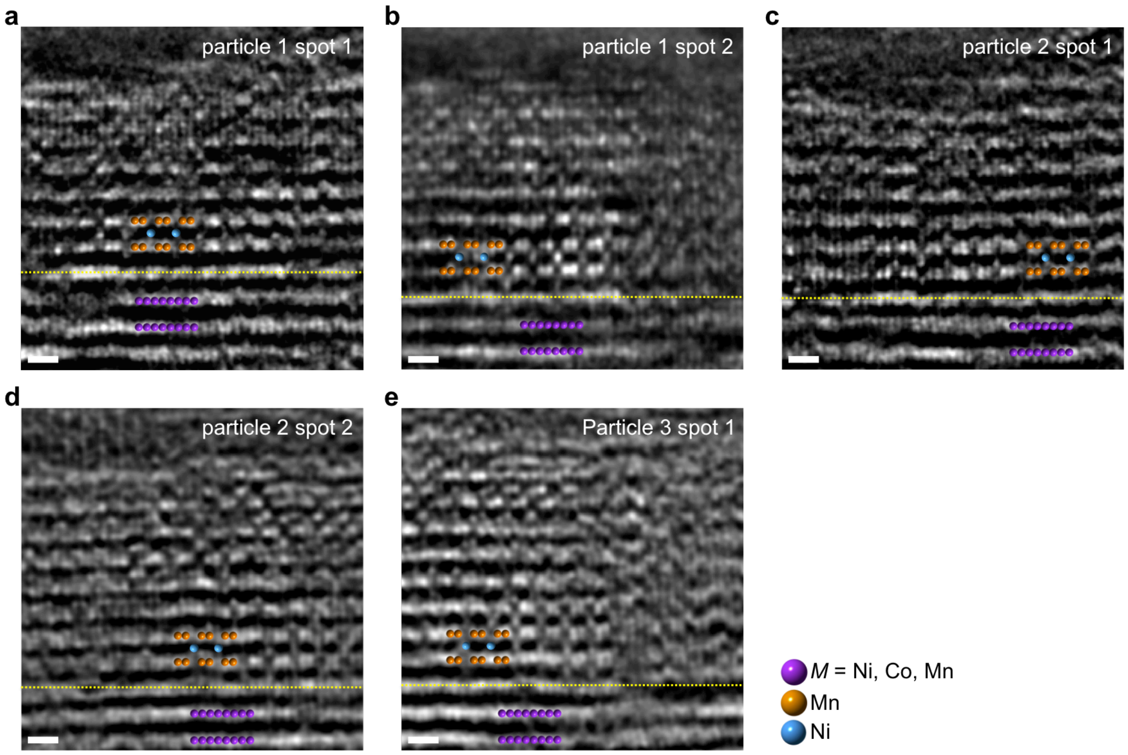

Supplementary Figure 15. Structure analyses of surface-modified sample from multiple spots and samples after the first charge. a-e, HAADF-STEM images of the surface-modified electrode projected along the monoclinic [100] direction attained from different spots of three particles. These images are consistent with the one in Figure 4b, validating the effect of the modified surface structure. Scale bars, 5 Å.

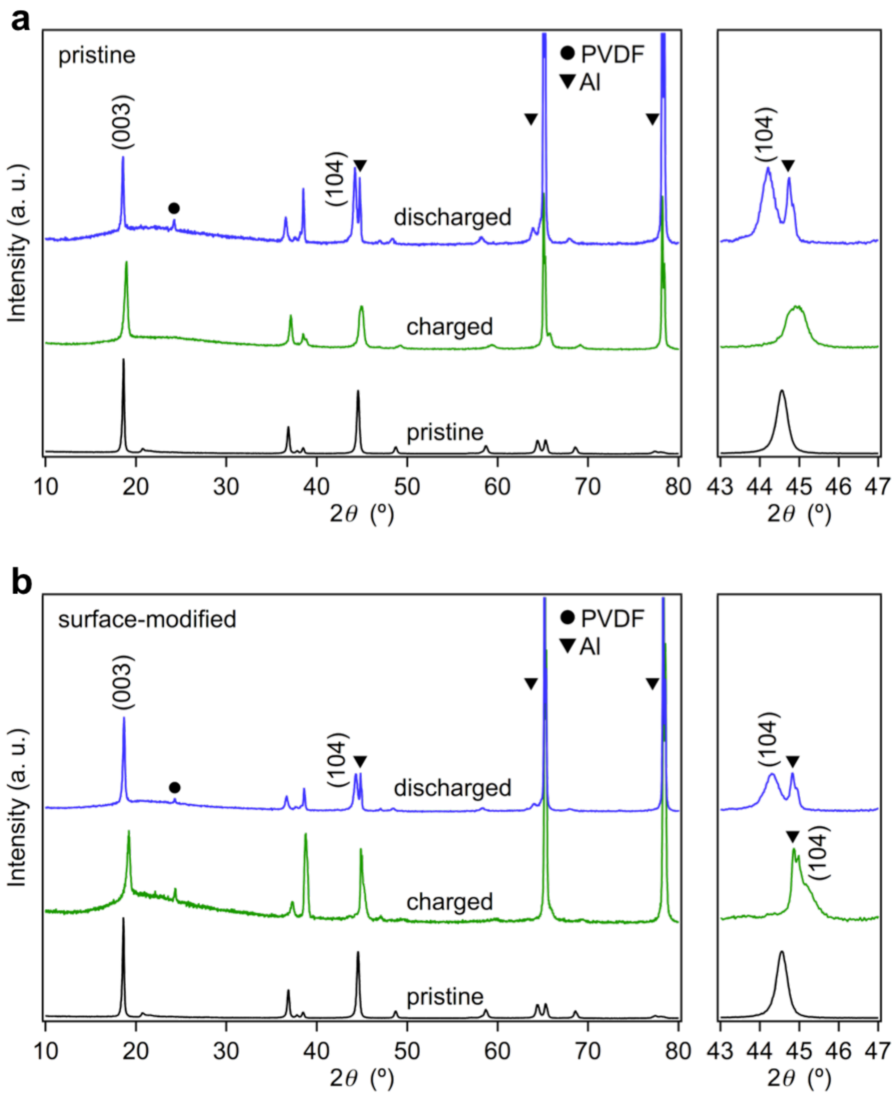

Supplementary Figure 16. XRD characterization during the first cycle . a, b, Ex-situ XRD profiles of (**a**) the pristine electrode and (**b**) the surface-modified electrode with 5 wt% Li_2MnO_3. The lower intensity ratio of $I_{(104)}/I_{(003)}$ of the surface-modified electrode after the charge-discharge cycle reflects suppressed phases transitions by the surface modification.

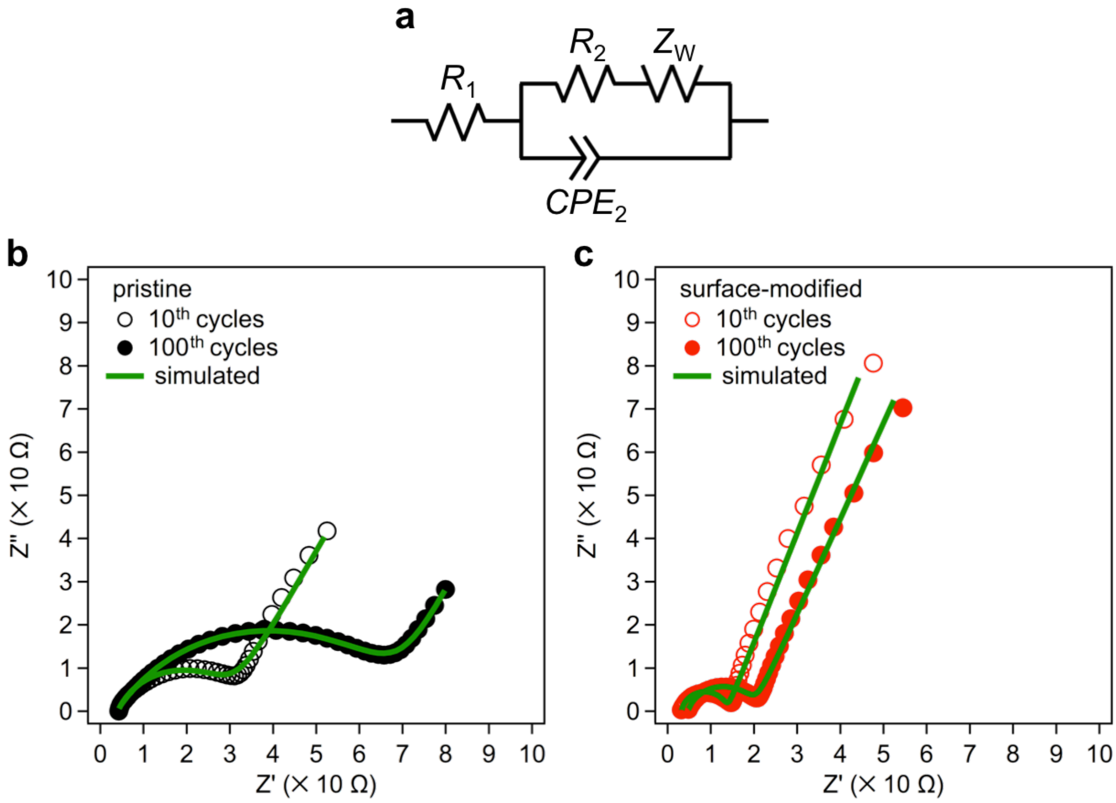

Supplementary Figure 17. Interfacial resistances of pristine and surface-modified electrodes. a, An equivalent circuit used for the fitting. **b, c,** Nyquist plots and simulated curves of (**b**) the pristine electrode and (**c**) the surface-modified electrode with 5 wt% Li_2MnO_3 after 10 and 100 cycles.

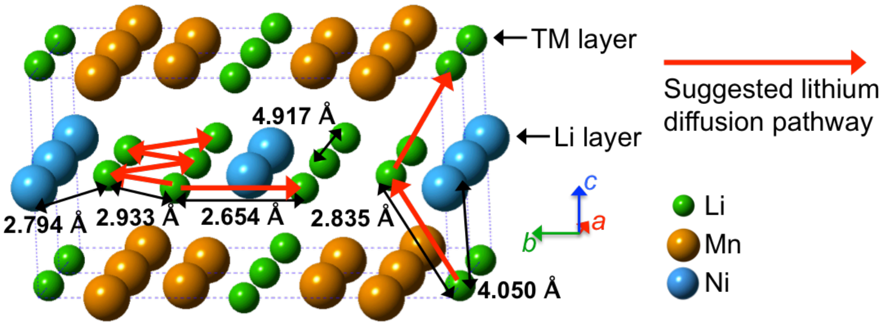

Supplementary Figure 18. Suggested Li ion diffusion pathways in the modified surface. Crystal structure of the modified surface, distances between neighboring atoms, and possible Li ion diffusion pathways. The blue dotted line indicates the unit cell. For clarity, oxygen is not displayed.

Supplementary Table 1. Intensity ratio between the superlattice and (003) peaks in Supplementary Fig. 3.

wt% of Li_2MnO_3	$I_{(superlattice)}/I_{(003)}$
0	0.031
1	0.031
5	0.032
20	0.038

Supplementary Table 2. Quantitative values of circuit components in EIS analyses shown in Supplementary Fig. 17.

	Pristine				Surface-modified			
	R_1 (Ω)	R_2 (Ω)	C_2 (F)	Component	R_1 (Ω)	R_2 (Ω)	C_2 (F)	Component
10th cycle	3.9	29.2	2.7×10^{-6}	Interface	3.2	11.0	1.1×10^{-6}	Interface
100th cycle	3.7	68.2	1.9×10^{-6}	Interface	4.6	15.9	1.6×10^{-6}	Interface

Supplementary Table 3. Crystal structure information used in Supplementary Fig. 18.

Atom	Site	*x*	*y*	*z*
Li(1)	2*b*	0	0.5	0
Li(2)	4*h*	0	0.656	0.5
Ni	2*c*	0	0	0.5
Mn	4*g*	0	0.1663	0
O(1)	4*i*	0.2178	0	0.2253
O(2)	8*j*	0.2537	0.322	0.2237

Note: space group *C*2*m*, a = 4.9167 Å, b = 8.5069 Å, c = 5.0099 Å, α = 90°, β = 109.373°, γ = 90°. Li in 2*c* sites of (ref . 1) is replaced by Ni.

Supplementary Note 1. Crystal structures of conventional layered and Li-rich layered oxides.

The Li-rich layered oxide ($0.5Li_2MnO_3$-$0.5LiNi_{0.44}Mn_{0.32}Co_{0.24}O_2$ or $Li_{1.2}Mn_{0.528}Ni_{0.176}Co_{0.096}O_2$) investigated in this study is a solid solution of two phases, $LiMO_2$ (M = Ni, Co, Mn) and Li_2MnO_3. Supplementary Fig. 1 shows the crystal structures of $LiMO_2$ and Li_2MnO_3. The layered $LiMO_2$ belongs to the space group $R\bar{3}m$ in the rhombohedral symmetry. In its structure, both Li and M occupy the octahedral sites in a cubic close-packed framework of oxygen atoms[2] and the Li and M layers are alternately repeated (Supplementary Fig. 1a). By contrast, in monoclinic Li_2MnO_3 (space group $C2m$), one-third of the octahedral positions in the TM planes are occupied by Li to form ordered $Li_{1/3}Mn_{2/3}$ slabs[3] (Supplementary Fig. 1b). It is well known that large charge-discharge capacities of Li-rich layered oxides are associated with activation of Li_2MnO_3 during the first charging process[4,5]. It has also been reported that the surface region of Li_2MnO_3 is actively reconstructed and converted to a high performance phase[6]. Based on these considerations, Li_2MnO_3 was chosen as a coating material in our study.

Supplementary Fig. 2a shows a XRD pattern of the Li-rich oxide material ($0.5Li_2MnO_3$-$0.5LiNi_{0.44}Mn_{0.32}Co_{0.24}O_2$). The observed diffraction peaks were well indexed based on the hexagonal unit cell of the layered structure with the space group symmetry $R\bar{3}m$ (No. 166)[4] except for superlattice peaks around 2θ = 20-25°. The superlattice peak appeared broadened, especially for the $(1/3, 1/3, l)_{\mathrm{hex}}$ planes, reflecting a stacking fault of the $[\sqrt{3}a_{\mathrm{hex}} \times \sqrt{3}a_{\mathrm{hex}}]R30°$-type superlattice planes along the c-axis[3,4]. The TEM image confirmed the well-developed layered structure (Supplementary Fig. 2b). The stacking fault was revealed in the FFT pattern obtained (red arrows in Supplementary Fig. 2c) from the TEM image.

Supplementary Note 2. XPS and XRD analyses before and after surface modification.

The surface-modified electrode displays a high Ni content at the surface region (Supplementary Fig. 6). Cho *et al*. reported that Ni ions (Ni^{2+}) reduced by the substitution of Mn^{4+} could easily migrate to the surface[7]. The Ni diffusion can also be induced by a heat treatment[8,9] due to its high diffusivity. Since the oxidation state of Ni was consistent before and after the surface modification (Supplementary Fig. 7), the Ni diffusion is driven mainly by the heat treatment.

After the surface modification, the lattice parameters became slightly smaller (pristine: a_{hex} = 2.802(3) Å, c_{hex} = 14.174(1) Å, surface-modified with 5 wt% Li_2MnO_3: a_{hex} = 2.801(1) Å, c_{hex} = 14.171(7) Å). Nonetheless, after the surface modification, the full width at half maximum of the (003) peak did not increase (pristine: 0.241°, surface-modified with 5 wt% Li_2MnO_3: 0.224°) and no impurity phases were observed. These results indicate that the Ni diffusion during the heat treatment proceeds in a very stable manner without harming the original crystal structure through the well-connected crystal structure between the host and the surface.

Supplementary Note 3. Electrochemical properties before and after surface modification.

The electrode with 20 wt% Li_2MnO_3 exhibited worse rate capability compared with the pristine electrode (Supplementary Fig. 8). As no impurity phases were observed for this modified sample, the deteriorated rate capability is presumably due to lower crystallinity and reduced uniformity of the surface layer, which hinders Li ion diffusion at the electrode/electrolyte interface, although further investigation is needed for clarification.

The charge-discharge profiles of the pristine electrode clearly showed voltage fading, in particular, around 3.0 V in the discharge process (Supplementary Figs. 12a and 13a). Meanwhile, the surface modification significantly reduced the voltage fading (Supplementary Figs. 12b and 13b), reconfirming the critical role of the modified surface in the suppression of phase transitions. This effect was also reflected in dQ/dV plots (Supplementary Fig. 14).

Supplementary Note 4. EIS analyses during electrochemical cycling.

All the impedance plots present one semicircle and thus can be analyzed quantitatively using the same equivalent circuit (Supplementary Fig. 17a). Empirically, capacitances of the bulk, grain boundary, and interface regions for ionic conduction are on the order of 10^{-12}, 10^{-9}, and 10^{-6} F, respectively[10]. According to our simulation, the capacitance components of the semicircles are on the order of 10^{-6} F (Supplementary Table 2), hence the relevant process is the charge-transfer at the electrode-electrolyte interface. The surface-modified electrode exhibited far lower interfacial resistance compared with that of the pristine electrode, which may be explained by the fact that the enhanced structural stability by the surface modification facilitates Li ion transfer at the interface and mitigates side reactions with the electrolyte solution. The enhanced interfacial stability is also reflected in a much smaller change in the interfacial resistance after 100 cycles. The deteriorated Li ion transfer kinetics for the pristine electrode would cause the voltage polarization during cycling. The well-defined ionic channels (Supplementary Fig. 18 and Supplementary Table 3) in the modified surface region are expected to play a role in the facile Li ion transfer at the interface.

References

1. Lee, S. *et al.* Antiferromagnetic ordering in Li_2MnO_3 single crystals with a two-dimensional honeycomb lattice. *J. Phys.: Condens. Matter* **24,** 1-9 (2012).

2. Armstrong, A. R., Bruce P. G. Synthesis of layered $LiMnO_2$ as an electrode for rechargeable lithium batteries. *Nature* **381,** 499-500 (1996).

3. Yabuuchi, N. *et al.* Detailed studies of a high-capacity electrode material for rechargeable batteries, Li_2MnO_3-$LiCo_{1/3}Ni_{1/3}Mn_{1/3}O_2$. *J. Am. Chem. Soc.* **133,** 4404-4419 (2011).

4. Thackeray, M. M. *et al.* Li_2MnO_3-stabilized $LiMO_2$ (M = Mn, Ni, Co) electrodes for lithium-ion batteries. *J. Mater. Chem.* **17,** 3112-3125 (2007).

5. Armstrong, A. R. *et al.* Demonstrating oxygen loss and associated structural reorganization in the lithium battery cathode $Li[Ni_{0.2}Li_{0.2}Mn_{0.6}]O_2$. *J. Am. Chem. Soc.* **128,** 8694-8698 (2006).

6. Taminato, S. *et al.* Highly reversible capacity at the surface of a lithium-rich manganese oxide: a model study using an epitaxial film system. *Chem. Commun.* **51,** 1673-1676 (2015).

7. Cho, Y., Oh P., Cho J. A new type of protective surface layer for high-capacity Ni-based cathode materials: nanoscaled surface pillaring layer. *Nano Lett.* **13,** 1145-1152 (2013).

8. Gu, M. *et al.* Conflicting roles of nickel in controlling cathode performance in lithium ion batteries. *Nano Lett.* **12,** 5186-5191 (2012).

9. Zheng, J. *et al.* Mitigating voltage fade in cathode materials by improving the atomic level uniformity of elemental distribution. *Nano Lett.* **14,** 2628-2635 (2014).

10. Felice, C. J. *et al.* Impedance microbiology: quantification of bacterial content in milk by means of capacitance growth curves. *J. Microbiol. Meth.* **35,** 37-42 (1999).
